# Sexual function and sexual activity in young total hip arthroplasty Chinese patients: A retrospective cohort study

**DOI:** 10.3389/fsurg.2022.960721

**Published:** 2023-01-05

**Authors:** Daishui Yang, Jie Zhang, Kexin Zhang, Yanlin Zhou, Xiao Peng, Ling Wang, Tang Liu

**Affiliations:** ^1^ Department of Orthopaedics, The Second Xiangya Hospital, Central South University, Changsha, China; ^2^ Clinical Nursing Teaching and Research Section, The Second Xiangya Hospital of Central South University, Changsha, China; ^3^ Operation Room, The Second Xiangya Hospital of Central South University, Changsha, China; ^4^ Department of Psychology, School of Public Health, Southern Medical University, Guangzhou, China; ^5^ Department of Orthopaedics, Changsha Hospital of Traditional Chinese Medicine, Changsha Eighth Hospital, Changsha, China; ^6^ Florence Nightingale Faculty of Nursing, Midwifery & Palliative Care, King’s College London, London, United Kingdom

**Keywords:** total hip arthroplasty, sexual function, sexual activity, sexual-related attitude, young patients

## Abstract

**Introduction:**

As an essential component of quality of life, there is limited evidence of sexual function (SF), especially for young patients, before and after total hip arthroplasty (THA). This study aims to enhance the understanding of SF status and assess patient perspectives before and after THA.

**Methods:**

A total of 109 patients who received THA were enrolled into our retrospective cohort study. To assess the SF status before and after THA, patients were required to fill out a standardized SF questionnaire [female sexual function index (FSFI) or brief sexual function inventory (BSFI) for males] and a specifically designated questionnaire regarding perspectives toward sexual activity and attitudes to sexual-related information.

**Results:**

Total average scores of both FSFI and BSFI were higher post-THA. For female patients, the FSFI scores were significantly higher in the domain of desire, orgasm, and satisfaction (*p* < 0.05). For male patients, the BSFI scores were also improved in the sex drive and satisfaction domain post-operation (*p* < 0.05). A large proportion of the patients (64.22%) reported difficulty in sexual activity preoperatively, primarily due to restricted motion (82.86%) and hip pain (74.29%). After a successful procedure, there was a reduction in difficulty in patients’ sexual activity post-THA (39.45%), mainly attributed to less pain (72.09%) and greater mobility (79.07%). In addition, subgroup analysis results indicated that gender and severity of hip stiffness and pain were crucial factors that could affect the patient's SF status. Furthermore, the majority of patients reported that they desired information concerning sexual activity, but only 12.84% of patients were informed well. Patients’ preferred channels to acquire sexual-related information was a booklet (65.59%, *n* = 61), followed by informing a surgeon and a nurse. The most concerning questions regarding the sexual activity of patients were the time to recovery (90.32%) and safe postures (76.34%).

**Conclusion:**

The majority of men and women who underwent THA reported their SF status return to baseline or have improved, mainly attributable to less pain and greater mobility. Age and severity of hip pain/stiffness were the factors that could affect patients’ SF status. Sexual education for young THA patients is needed due to the lack of related information during hospitalization.

## Introduction

Among hip disease patients, chronic hip pain and restricted movement heavily affect their daily activities. Total hip arthroplasty (THA), as an effective treatment to improve patient-focused quality of life (QoL), has been extensively applied to relieve pain and restore essential hip function in patients affected with hip disease [e.g., osteoarthritis (OA), osteonecrosis of the femoral head (ONFH), and developmental dysplasia of the hip] (1, 2). More than one million total joint arthroplasties have been performed every year worldwide, and the number has been increasing among middle-aged adults (3). The effects of THA on the general QoL have been well documented in young patients to reduce pain and joint stiffness and improve function satisfaction in daily activities (4–6). While sexual function (SF) is a valued component of patient-reported QoL and is positively associated with better overall health in young patients’ life, limited understanding exists of postoperative young hip disease patients around the world. Although there are several scales being applied in clinical practice to evaluate the general condition of patients during hospitalization, such as the SF-36 questionnaire, Harris hip score, and the Oxford Hip Score, few have incorporated focused questions regarding patients’ SF (7–9).

Previous emerging research has demonstrated that patients with hip diseases have lower levels of sexual activity, and a certain percentage of them resumed sexual function with THA. Issa et al. systematically reviewed 10 eligible reports yielded between 1970 and 2015, and the outcomes suggested that THA was associated with better sexual activity in 1,694 patients who had an average age of 57 years (10). However, the majority of patients in the included studies were over 60 years old, whereas only two surveyed young patients’ SF around the world. Nunley et al. investigated the impact of THA on SF in young, active patients and reported that most young patients return to their baseline or higher level of sexual activity after THA (11). However, the subdomain of sexual function (such as sexual desire, arousal, and orgasm) among both males and females have never been well investigated among pre- and post-THA patients. Hence, it is meaningful to investigate the specific components of SF and THA treatment-induced SF changes among young patients.

Several validated instruments have been widely used to examine SF or diagnostic sexual dysfunction in either males or females, such as the Brief Index of Sexual Functioning for Women (BISF-W), the Changes in Sexual Functioning Questionnaire (CSFQ), the Female Sexual Function Index (FSFI), International Index of Erectile Function (IIEF) and the Brief Sexual Function Inventory (BSFI) (12, 13). FSFI and BSFI are two validated questionnaires that have been used frequently to measure sexual function. The former is a brief, self-report questionnaire that provides scores on five domains of SF status for females, including desire, arousal, lubrication, orgasm, pain, and satisfaction (14, 15). The latter is a short, validated questionnaire for males that addresses sex drive, erectile and ejaculatory function, and overall sexual satisfaction (16). Hence, we adopted the validated questionnaire FSFI and BSFI as a tool to evaluate the SF status of young patients pre- and post-THA. In addition, a specifically designated questionnaire was adopted to acquire detailed information on young patients’ attitudes to sexual-related information.

This study aimed to (1) investigate SF scores and the proportion of sexual dysfunction pre- and post-THA; (2) evaluate the alterations of sexual activity experience following THA; (3) uncover the specific factors/associations affecting patients’ SF; and (4) explore young patients’ attitudes/perspectives toward sexual-related information.

## Methods

This was a quantitative study to assess patients’ SF score acquired from FSFI for women and BSFI for men. Meanwhile, it was also a qualitative study to investigate patients’ sexual activity and attitudes toward sexual-related information *via* an online semistructured questionnaire in the same patient cohort.

### Study design

We performed a retrospective cohort study of hip disease patients who underwent THA between 2013 and 2020 in two local hospitals. Patients’ postoperative information was collected using an electronic medical record system and Internet questionnaires filled by participants from October 2018 to October 2020. Inclusion criteria were (1) married or living with someone at the time of onset of hip symptoms; (2) age ≤50 years but ≥20 years when had undergone THA; (3) 6 months to 5 years after THA. Those who met the exclusion criteria will be ruled out: (1) wound infection, prosthesis loosening, and joint dislocation postoperative; (2) severe diseases, such as malignant tumors, and serious heart diseases pre- and post-operation.

### Data collection

The research team included two doctors, three nurses, and two translators who had collected the basic information of 1,186 patients from the electronic medical record system. After screening, 727 (61.3%) patients were more than 50 years old, 117 (9.9%) patients had other comorbidities that could affect their SF, and 342 (28.8%) patients met the inclusion and exclusion criteria mentioned above. Our team contacted these patients by telephone to explain the purpose of this study, followed the voluntary principle, and explained that patients’ information would not be used for commercial purposes. Of these patients, 67 could not be contacted or had communication barriers, 145 refused to participate, and 130 patients signed the electronic consent form to study participation. We sent the web link address of the questionnaire to them, and finally, 109 patients completed the questionnaire.

### Instruments

The questionnaire consisted of four domains: demographic characteristics and medical history, the Chinese version of the validated SF questionnaire (FSFI and BSFI for women and men, respectively), sexual activity pre- and post-THA, and patients’ attitudes to sexual activity-related information. The first domain mainly collected patients’ general information, including self-reported age, sex, residence, etiology, primary diagnosis, duration of hip disease, time of surgery, comorbidities, the severity of pain, and stiffness of hip before and after surgery (the scores of hip pain and stiffness, ranging between 0 and 10, where 0 represents no problems and 10 extreme pain and restricted movement) (Appendix 1, part 1).

The second domain aimed to acquire detailed information on patients’ SF status pre- and post-THA. The available Chinese version of the FSFI consists of 19 questions and covers a range of six dimensions of SF, taking approximately 10–15 min to complete (17). The BSFI is a self-report measure of SF and consists of five dimensions. The original English version of the BSFI was translated into a simplified Chinese version by experts who are bilingual in both Chinese and English. Then, it was back-translated by a professional translator. Next, the back-translation was compared with the original BSFI and some revisions were made to ensure that the translated version was highly consistent with the original BSFI on each item. The domain score of FSFI and BSFI were calculated by the specific formula and higher scores mean better function (Appendix 1, part 2).

The third and fourth domains of the questionnaire were designed by referring to the literature of Currey (18), Nunley et al. (11), and Yoon et al. (19). The third domain mainly addressed sexual activity before and after THA: status of patients’ sexual activity before and after THA, alterations of sexual activity after the operation, and reasons for sexual activity alterations (Appendix 1, part 3). Questions in the fourth domain were related to patients’ attitudes to sexual-related information: reception of information related to sexual activity, ways of obtaining sexual-related information pre- and post-THA, favorite channels to obtain related information, and the most concerned questions post-THA (Appendix 1, part 4).

### Data analysis

The data analysis was performed after all questionnaires had been fulfilled. The individual domain score of the FSFI and BSFI was collected by designated computational formula. It should be noted that the total scale score of FSFI was obtained by adding each individual domain score, and the total scale score of BSFI was calculated by taking the average of each subitem. Data aggregation and analysis were performed using SPSS 27.0 (SPSS Inc., United States), and values were expressed as average ± standard deviation (SD). Independent-sample T-test, paired-sample T-test, and chi-square tests were performed to compare the mean value between groups. Binary logistic regression was performed in subgroup analyses. The threshold of significance retained was *p* < 0.05.

### Ethical considerations

Ethical approval was obtained from the Medical Ethics Committee at Central South University. All participants were told their sensitive information would be kept absolutely confidential and no individual information could be identifiable. Only authors have access to look through participants’ original data and electronic consent forms.

## Results

### Demographic

The clinical and demographic characteristics of patients are summarized in Table 1. A total of 109 participants (53 women and 56 men) met inclusion/exclusion criteria and completed the baseline questionnaires. The mean age of female and male patients was 40.91 ± 5.41 and 42.21 ± 5.03 years, respectively. Participants were mainly diagnosed with ONFH (54.13%), OA (21.10%), and others (e.g., ankylosing spondylitis, developmental dislocation of the hip, and rheumatoid arthritis). The hip stiffness and pain score of women caused by hip pathology were higher compared to men both pre- and post-operation.

**Table 1:** Demographic characteristics of patients.

Characteristic	No. (%)		*p*-value
	Female	Male	
No. of patients	53	56	
Age, mean (SD)	40.91 (5.41)	42.21 (5.03)	0.19
Rural/city	19/34 (35.85/64.15)	23/33 (41.07/58.93)	0.58
Primary diagnosis			
ONFH	27 (50.94)	32 (57.14)	0.70
OA	11 (20.76)	12 (21.43)	
Others^a^	15 (28.30)	12 (21.43)	
Duration of disease (years)			
<5	21 (39.62)	23 (41.07)	0.91
5–10	20 (37.74)	19 (33.93)	
>10	12 (22.64)	14 (25.00)	
Comorbidities			
Hypertension	3 (5.67)	5 (8.93)	
Diabetes	3 (5.67)	3 (5.36)	
Pre-THA, mean (SD)			
Pain score	7.42 (1.13)	6.98 (1.37)	0.08
Stiffness score	7.72 (1.12)	7.18 (1.60)	<0.05
Post-THA, mean (SD)			
Pain score	2.96 (1.52)	2.45 (1.06)	<0.05
Stiffness score	3.42 (1.52)	3.05 (1.48)	0.21

SD, standard deviation; ONFH, osteonecrosis of femoral head; OA, osteoarthritis; pre-THA, before total hip arthroplasty; post-THA, after total hip arthroplasty.

^a^ Others include developmental dysplasia of the hip and rheumatoid arthritis.

### Status of sexual function

Paired analyses of SF scores before and after THA are provided in Table 2. Overall scores of standard questionnaires in post-THA patients had significantly increased compared to pre-THA matched peers. It was noted that preoperative patients averaged a total FSFI score of 19.61 ± 4.97, as compared to 22.1 ± 4.53 after surgery (*p* < 0.05, Table 2). Results showed that significant improvement was observed in the domain of sexual desire, orgasm, and satisfaction after THA operation. It seems likely that pain during sexual intercourse improved postoperatively, but there was no significant statistical difference (*p* = 0.07) between pre-THA (3.78 ± 1.06) and post-THA (4.05 ± 0.81). Obviously, no noticeable differences were observed in sexual arousal and lubrication. The average total BSFI score reported by males post-THA was 2.72 ± 0.56, which was also significantly improved than the value for pre-THA 2.47 ± 0.61 (*p* < 0.05, Table 2), especially in the domain of sex drive and satisfaction. However, there was no evident change in the rest domain of BSFI. For both FSFI and BSFI, higher scores indicated better sexual function.

**Table 2:** FSFI and BSFI domain and total scores (average ± SD) for our patient cohort.

Domain	FSFI score, mean (SD)		*p*-value	Domain	BSFI score, mean (SD)		*p*-value
	Pre-THA	Post-THA			Pre-THA	Post-THA	
Total	19.61 (4.97)	22.10 (±4.53)	<0.05	Total	2.47 (0.61)	2.72 (0.56)	<0.05
Desire	2.31 (1.15)	2.92 (1.12)	<0.05	Sex drive	1.87 (0.85)	2.25 (0.83)	<0.05
Arousal	3.84 (0.94)	3.85 (0.93)	0.97	Erections	2.78 (0.65)	2.71 (0.67)	0.37
Lubrication	3.84 (0.92)	3.96 (0.99)	0.37	Ejaculation	3.03 (0.78)	3.19 (0.76)	0.11
Orgasm	3.33 (1.04)	3.68 (1.01)	<0.05	PA	2.85 (0.59)	2.88 (0.55)	0.68
Satisfaction	2.51 (0.98)	3.65 (0.91)	<0.05	Satisfaction	1.82 (1.03)	2.57 (0.93)	<0.05
Pain	3.78 (1.06)	4.05 (0.81)	0.07				

FSFI, female sexual function index; BSFI, brief sexual function inventory; SD, standard deviation; pre-THA, before total hip arthroplasty; post-THA, after total hip arthroplasty; PA, problem assessment.

### Patient-reported sexual function

Specific sexual activity questionnaires were designed to further investigate detailed information about sexual activity status before and after the surgery as well as reasons for subjective changes postoperatively. As shown in Table 3, 64.22% (*n* = 70, 33 males and 37 females) of patients reported difficulty performing sexual activities before THA, mainly attributable to their stiffness (82.86%), hip pain (74.29%), and muscle weakness (31.43%). There was a marked reduction in the percentage of patients (46.79%) with abnormal sexual activity following THA. Of the patients, 39.45% (*n* = 43, 24 males and 19 females) reported obtaining better sexual experience, citing greater mobility (79.07%) and/or less hip pain (72.09%) as the primary cause. In contrast, only 11.01% of the patients (*n* = 12, 7 males and 5 females) reported worsened sexual experience generally due to hip discomfort (58.33%) and concerned hip dislocation (75.00%) after surgery. The remainder (49.54%) stated that their sexual activities remained unchanged pre- and post-THA.

**Table 3:** Sexual activity before and after THA.

	Number of patients (%)	Females: number (%)	Males: number (%)
Sexual activity before THA			
Status of sexual activity			
Normal	39 (35.78)	16 (30.19)	23 (41.07)
Abnormal	70 (64.22)	37 (69.81)	33 (58.93)
Reason for sexual difficulty			
Hip pain	52 (74.29)	28 (75.68)	24 (72.73)
Restricted motion	58 (82.86)	31 (83.78)	27 (81.82)
Muscles weakness	22 (31.43)	12 (32.43)	10 (30.30)
Sexual activity after THA			
Status of sexual activity			
Normal	58 (53.21)	26 (49.06)	32 (57.14)
Abnormal	51 (46.79)	27 (50.94)	24 (42.86)
Change of sexual activity			
Worse	12 (11.01)	5 (9.43)	7 (12.50)
Hip discomfort	7 (58.33)	3 (60.00)	4 (57.14)
Concerned dislocation	9 (75.00)	4 (80.00)	5 (71.43)
Other reasons	3 (25.00)	1 (20.00)	2 (28.57)
Same	54 (49.54)	29 (54.72)	25 (44.64)
Better	43 (39.45)	19 (35.85)	24 (42.86)
Less pain	31 (72.09)	13 (68.42)	18 (75.00)
Greater mobility	34 (79.07)	16 (84.21)	18 (75.00)
Muscles strength enhancement	16 (37.21)	5 (26.32)	11 (45.83)
Other reasons	6 (13.95)	2 (10.53)	4 (16.67)

THA, total hip arthroplasty.

### Patient-reported attitude

Table 4 provides a summary of patients’ responses to the questionnaire. Preoperatively, a large proportion of patients indicated that they desired sexual activity-related information, while only 16.51% of patients reported they have a deep understanding of the impact of their hip disease on sexual activity. Affected sexual activity was constituted as one of the essential factors in the decision to undergo the operation by 18.35% of the patients. Of those who managed to obtain sexual-related information (18 patients), the most common source of information was mainly the Internet (66.67%), followed by medical staff (33.33%). When asked about receiving related information during hospitalization after the operation, a great proportion of patients felt they received inadequate information about sexual activity. Most patients wish to obtain information from a booklet (65.59%) and a surgeon (48.39%). Safe positions (71 patients, 76.34%) during sexual intercourse and the first time (84 patients, 90.32%) for sexual activity were the most common concern after surgery. However, only 11.01% of the patients reported that they were informed well about the time of recovery activity, and 4.59% received information about safe postures for sexual activity.

**Table 4:** Patient's attitude to sexual activity-related information before and after THA.

Question No. (see Appendix IV)	Summary of question	Summary of responses
Pre-THA period		
1	Did you desire for sexual activity-related information before THA?	Y: 88 (80.73%) N: 21 (19.27%)
2	Did you realize the impact of THA on sexual activity?	Y: 18 (16.51%) N: 91 (83.49%)
(Q2 yes) 3	What channels did you acquire information on this subject?	Internet: 12 (66.67%) Doctor: 6 (33.33%)
4	Did you decide to THA because of affected sexual activity?	Y: 20 (18.35%) N: 89 (81.65%)
Post-THA period		
5	Did you receive information about sexual activity after THA?	Y: 14 (12.84%) N: 95 (87.16%)
6	Did you desire for sexual activity-related information after THA?	Y: 93 (85.32%) N: 16 (14.68%)
(Q6 yes) 7	Which channels did you want to acquire sexual activity-related information most?	Booklet: 61 (65.59%) Doctor: 45 (48.39%) Nurse: 36 (38.71%)
(Q6 yes) 8	What information did you want to obtain most?	A: 84 (90.32%) B: 71 (76.34%)
9	Were you informed of the time of recovery activity?	Y: 12 (11.01%) N: 97 (88.99%)
10	Were you informed of safe postures for sexual activity?	Y: 5 (4.59%) N: 104 (95.41%)

THA, total hip arthroplasty; Y, yes; N, no; A, time of recovery sexual activity; B, safe postures of sexual activity.

### Subgroup analyses

As shown in Table 5, older patients were more prone to suffer from sexual activity difficulties pre-operation (OR: 1.12, 95% CI: 1.02–1.24); the difference did not exist after a successful procedure (*p* > 0.05). In addition, sexual activity in the female population was approximately more likely to be affected by their hip disease, especially before surgery, but the difference was not statistically significant. Further analysis was performed on hip pain and stiffness, respectively. Before treatment, there were clearly negative associations between SF scores and severity of joint stiffness, indicating worsening SF in patients with severe joint stiffness. Postoperatively, patients with hip pain relief were positively predictable to better SF overall score (OR: 2.05, 95% CI: 1.32–3.20). In addition, increased hip joint mobility was also beneficial to SF score improvement (OR: 1.87, 95% CI: 1.28–2.73), which indicates better sexual experience (Table 5).

**Table 5:** Subgroup-specific association of sexual function with age, gender, joint pain, and stiffness.

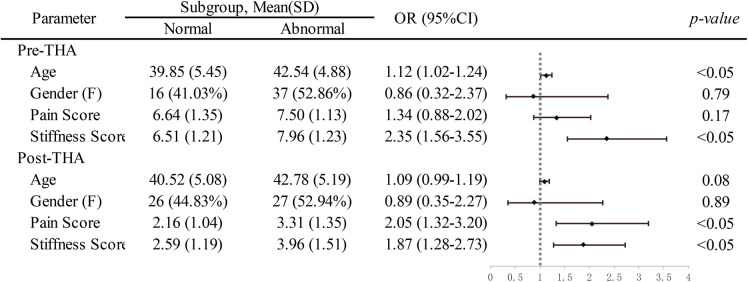

OR: odds ratio; 95%CI: 95% confidence interval; F: female.

## Discussion

Total hip arthroplasty has proven to be an effective surgical intervention to ameliorate the adverse effects of hip diseases in young patients (20, 21). Sexual life is an essential component determining QoL and satisfaction, especially in the young group, and restoring the ability to engage in sexual activity may actually be a critical goal after the operation (6, 22). However, until now, there has been relatively scant work on examining the specific domains of SF and the changes in sexual activity post-THA in young, active patients afflicted with hip disease. The majority of previously published studies mainly adopted interviews (face-to-face or telephone) or self-designed questionnaires to investigate sexual activity in patients, focused on small cohorts and patients greater than 50 years old. FSFI and BSFI are two routine questionnaires for standardized assessment of SF, which have been frequently used in other fields, such as evaluating SF after spinal and urological surgery. The purpose of the current study was thus to use validated FSFI and BSFI to evaluate SF, document subjective changes in sexual activity and reasons for such alterations by comparing pre-and post-THA in young patients, and obtain patients’ attitudes to sexual-related information.

To the best of our knowledge, our study adds novel results by adopting standardized questionnaires to examine specific dimensions of SF and detailed alterations of sexual activity postoperatively and attempts to identify specific factors affecting patients’ SF. Before surgery, 64.22% of patients (37 women and 33 men) reported deterioration of SF attributed to their hip pathology, with the predominant cause being hip pain and restricted movement. Postoperatively, nearly half of patients had significantly higher scores of FSFI and BSFI, which indicated improved SF and enhanced quality of sexual life, mainly attributed to pain relief (72.09%) and greater mobility (79.07%). Our results were concordant with that found in previous literature. For Klit et al. the resumption of sexual activity in 18/39 female patients demonstrated the beneficial effect of THA (23). Similarly, Issa et al. systematically reviewed a total of 1,694 THA patients with age nearly 60 years and indicated that arthroplasty had a positive effect on hip pathology-induced sexual dysfunction. Although Wang et al. in research of 247 ONFH patients with an average age of 46.8 years, reported no significant difference in the effect on sexual function between pre-THA and post-THA, THA also has improved significantly sexual satisfaction degree and relationship with their partner (25).

Furthermore, we found that SF scores were closely associated with patient-reported joint pain and stiffness degree, which indicated that more severe hip pain and stiffness commonly led to worse sexual experiences. The finding agreed with previous studies that sexual activity was known to be strongly correlated to physical and emotional dissatisfaction (26–28). THA is a very successful procedure to ameliorate the adverse effects on physics and emotion in hip disease patients (28, 29). Predictably, reduced pain and increased range of motion were conducive to SF restoration.

Overall scores of SF in females and males were significantly improved after the operation (19.61 ± 4.97 vs. 22.10 ± 4.53; 2.47 ± 0.61 vs. 2.72 ± 0.56, *p* < 0.05). The increase in BSFI scores post-THA was additionally noted for the sex drive domain (1.87 ± 0.85 vs. 2.25 ± 0.83, *p* < 0.05) and satisfaction domain (1.82 ± 1.03 vs. 2.57 ± 0.93, *p* < 0.05). Interestingly, the erections domain (2.78 ± 0.65 vs. 2.71 ± 0.67, *p* = 0.37) had not obviously changed after the procedure, which is inconsistent with one previous finding. Nordentoft et al. investigated sexual activity as well as erectile function in 53 male patients with a mean age of 70.6 years. The data showed a negative impact on erectile function in elderly males who had undergone arthroplasty: 26.1% of patients lost normal erectile function, and only 6.7% regained better erections postoperatively (30). Similarly, Libman et al. compared the sexual adjustment of operation for rectal cancer vs. inguinal hernia repair in elderly males and found a similar reduction in erectile function in elderly males (31). These funding indicated that major operations around the pelvis could have a significantly greater risk of erectile dysfunction in the elderly male population. Our study demonstrated a positive effect on overall SF, and erectile function had not obviously improved or deteriorated postoperatively in young patients. It seems likely that THA, one type of peripelvic surgery, has no adverse effects on erectile function in the relatively young group.

While hip pain and joint stiffness are implicated as important etiologic factors in causing sexual dysfunction, other factors such as patients’ age had clinically important impacts according to our results. The proportion of patients who regained sexual activity decreased with higher age, which has been similarly noted by others (11). In addition, our results confirmed that preoperative sexual activity did not differ significantly between the female and male groups (*p* = 0.07). It has been previously reported that sexual difficulties were more common in female patients, usually due to hip pain and stiffness rather than a loss of libido (18, 32, 33), which is not consistent with what we found, probably attributed to our small cohort study.

In our study, it was observed that the provision of sexual-related information was explicitly inadequate, which was not in accordance with patients’ needs. This lack of information was a problem mentioned by previous researchers (18, 32, 34–37). Ugwuoke et al. carried out a study with 17 clinicians and 244 patients and found that over 90% of patients expected their surgeons to discuss sex with them after THA, whereas clinicians rarely raised the sexual-related subject (34). Similarly, in a systematic review of 16 articles, with a total of 2,391 patients considered, Neonakis et al. stated that patient education regarding the resumption of sexual activity was severely lacking and the majority of surgeons offered little or no information on those subjects (37). During consultations, surgeons should understand the benefit that THA conveys to the sexual function of young hip disease patients and strive to manage patients’ concerns. The sexual-related subject needs to be broached with patients and they need to be given clear and detailed information (26).

This study has several limitations. First, with an average follow-up time of 3.04 ± 1.15 years in participants, it is possible to cause recall bias when participants evaluate the effect of THA on their SF and sexual activity several years postoperatively. Second, this is a retrospective cohort study, and we can only confirm the presence of an association between THA and improved SF postoperatively, but not definitely causation. Third, due to the personal nature of the topic, many patients refused to fulfill the questionnaire. The low response rate and small sample size recruited from institutions may not be representative. Finally, we adopted the BSFI questionnaire to obtain more detailed information on SF status, but its reliability and validity have not been verified well in the Chinese population. Future research will have to include a prospective analysis of SF with a larger sample size recruited from different institutions, along with a control group of nonoperatively treated patients with matched hip disease patients. All these in a future study may provide invaluable information about SF by controlling for possible self-reporting bias.

## Conclusion

More than half of the patients from this study underwent sexual dissatisfaction due to hip pathology (hip pain, joint stiffness, and others). After THA surgery, we found that better or equal SF status existed in a high rate among young patients by using standard SF questionnaires. Clinically, young patients are willing to obtain sexual-related information, yet strictly limited education is provided, which warrant further providing sexual consultant. These findings present a rich understanding of young THA patients’ sexual function that has never previously been investigated that could contribute to meeting patients’ expectations for their quality of life in the future.

## Data Availability

The raw data supporting the conclusions of this article will be made available by the authors, without undue reservation.

## Appendix 1. Questionnaires

Part 1. Demographics:

1. Whta is your sex?  ➀ Male  ➁ Female
2. What was your age when you had total hip arthroplasty? _______ years old
3. Where do you live in?  ➀ Rural  ➁ City
4. What caused your hip pathology?
  ➀ Alcohol abuse  ➁ Steroid-induced  ➂ Other ______
5. What was your diagnosis prior to total hip arthroplasty?
  ➀ Osteonecrosis of femoral head  ➁ Hip arthritis ➂ Other____
6. How long did hip pathology last before operation?
  ➀ <5 years  ➁ 5-10 years  ➂> 10 years
7. When did you have the hip arthroplasty? _______ (day/month/year)
8. What comorbidities did you have before operation?
  ➀ Hypertension  ➁ Diabetes  ➂ Other  ➃ None
9. How would you rate the severity of pain/stiffness before operation?
  (0 = no pain/stiffness, 10 = extremely pain/stiffness)
  0 1 2 3 4 5 6 7 8 9 10 (score of hip pain)
  0 1 2 3 4 5 6 7 8 9 10 (score of hip stiffness)
10. How would you rate the severity of pain/stiffness after operation?
  0 1 2 3 4 5 6 7 8 9 10 (score of hip pain)
  0 1 2 3 4 5 6 7 8 9 10 (score of hip stiffness)

Part 2. Sexual function scale:

FSFI:https://www.nva.org/wp-content/uploads/2015/01/FSFI-questionnaire2000.pdf

BSFI:https://www.malefertility.com/pdf/Sexual%20Dysfunction%20Inventory.pdf

Part 3. Sexual activity questionnaire:

Before total hip arthroplasty

1. Did you have difficulty with sexual activity due to hip pathology?
  ➀ Yes (go to question No. 2)  ➁ No (go to question No. 3)
2. What was the reasons for the difficulty before operation? (at least one answer)
  ➀ Hip pain  ➁ Restricted motion  ➂ Muscles weakness ➃ other
  After total hip arthroplasty
3. Did you have difficulty with sexual activity after operation?
  ➀ Yes ➁ No
4. Did you have any alterations with sexual activity after operation?
  ➀ Worse (go to question No. 5)  ➁ Same  ➂ Better (go to question No. 6)
5. what was the reason for deterioration in the quality of sexual activity after operation? (at least one answer)
  ➀ Hip discomfort  ➁ Concerned dislocation  ➂ Other
6. what was the reason for improvement in the quality of sexual activity after operation? (at least one answer)
  ➀ Less pain  ➁ Greater mobility  ➂ Muscles strength enhancement  ➃ Other

Part 4. Patients perspectives' questionnaire:

1. Did you desire for sexual activity related information before operation?
  ➀ Yes  ➁ No
2. Did you realize the impact of total hip arthroplasty on sexual activity?
  ➀ Yes (go to question No. 3)  ➁ No
3. What channels did you acquire information on this subject? _______
4. Did you decide to total hip arthroplasty because of affected sexual activity?
  ➀ Yes  ➁ No
5. Did you receive information about sexual activity after operation?
  ➀ Yes  ➁ No
6. Did you desire for sexual activity related information after operation?
  ➀ Yes (go to question No. 7 and 8)  ➁ No
7. Which channels did you want to acquire sexual activity related information most? (at least one answer)
  ➀ Doctor  ➁ Nurse  ➂ Booklet  ➃ Other
8. What information did you want to obtain most? (at least one answer)
  ➀ time of recovery sexual activity  ➁ safe postures for sexual activity  ➂ Other
9. Were you informed of the time of recovery activity?
  ➀ Yes  ➁ No
10. Were you informed of safe postures for sexual activity?
  ➀ Yes  ➁ No
